# Supplementary material for: Are carbon emissions trading and green financial instruments synergistic? -Comprehensive quantitative research based on content analysis
Source: PLoS One. 2024 Mar 7;19(3):e0298601. doi: 10.1371/journal.pone.0298601 (PMC10919601; doi:10.1371/journal.pone.0298601)
Supplement: S1 Data — (ZIP) [file pone.0298601.s001.zip › Data/地级市Z_F工作报告.zip!/░▓╗╒/╗╞╔╜╩╨/└┤╫╘╣1⁄2╓┌║┼í╛┬φ┐╦╩2╛▌═°í┐_2009.docx]

各位代表：
    现在，我代表黄山市人民政府，向大会作政府工作报告，请予审议，并请市政协委员和其他列席人员提出意见。

    一、2008年工作回顾
    刚刚过去的2008年，是黄山发展进程中非同寻常的一年。面对严重的自然灾害和严峻的金融危机的冲击影响，我们坚持以党的十七大、十七届三中全会和胡锦涛总书记视察安徽重要讲话精神为指导，在省委、省政府和市委的坚强领导下，深入贯彻落实科学发展观，紧紧围绕建设现代国际旅游城市战略目标，克难而进，扎实工作，较好地完成了市五届人大一次会议确定的年度目标任务。全市实现生产总值249.9亿元，比上年增长12%；财政收入25.7亿元，增长26.9%。人均生产总值、财政收入占生产总值比重首次突破2000美元和10%，城镇居民人均可支配收入、农民人均纯收入分别达到12801元和5160元，增长12.7%和20.1%，连续5年实现两位数增长。成绩来之不易，发展令人振奋！
    第一，坚持抢抓机遇，加大项目建设力度，发展后劲进一步夯实。我们始终紧紧抓住全球经济变化和宏观政策调整所带来的发展机遇，把投资作为加快发展的第一拉动力。在实施“443”行动计划的基础上，作出建设现代国际旅游城市的战略部署，制定了“十大工程”的实施意见。在全省率先出台一系列抗风险、保增长的措施，及时谋划和编报了73个投资总额达1550亿元的重点项目，在19个领域共争取到新增国债资金1.95亿元。全年完成全社会固定资产投资266.7亿元，增长25.1%；省“861”和市级重点建设项目分别完成投资42.6亿元和171.7亿元。抢上了黄杭高铁、京福高铁和皖赣铁路扩能改造等国家重点建设项目，黄塔（桃）高速公路和黄山机场国际航站楼建设竣工，机场改造、宣黄高速、黄祁高速和新安江延伸段综合开发等“十大工程”项目全面开工。过去的一年，我市全社会固定资产投资连续第9年实现了20%以上的速度增长，连续第3年跨上了净增50亿元的台阶，特别是令全市人民欢欣鼓舞的三大铁路干线争取成功，黄山将迎来现代交通的高铁时代，这是我们抢抓机遇效果最好的一年，是争取项目投资最多的一年！
    第二，坚持好中求快，强化产业发展优势，经济实力进一步壮大。我们始终坚持工业经济支撑，突出旅游经济中心。积极应对金融危机挑战，全力“促一批企业上水平、保一批企业度难关、上一批企业添后劲、调一批企业优结构”，工业化率和工业对经济增长的贡献率分别提高了2.5、10.2个百分点。全市规模以上企业增加到390户，实现增加值60.1亿元，增长29.8%；完成技改投入32.5亿元，增长19.5%，高新技术产业实现总产值70亿元，增长36%，高新技术企业达到69家。全市园区完成投资44.1亿元，新增入园企业101户。面对罕见的自然灾害和低迷的市场环境，我们创新营销拓展客源市场，整治环境提升旅游品质，全年接待游客1801.3万人次，旅游总收入140.9亿元，增长17.3%和27.4%；入境游客81万人次，创汇2.2亿美元，增长18.2%和25.7%。黄山风景区进山人数达224.4万人，再创历史新高。扎实推进国家服务标准化试点城市建设，继续实施万村千乡市场工程，繁荣城乡市场，实现社会消费品零售总额90.4亿元，增长23.7%。全市金融机构贷款余额154.8亿元，增长21.4%，增幅高于全省平均水平。全市三次产业的结构已由上年的14.6∶37.1∶48.3，调整到13.7∶39.5∶46.8。过去的一年，我市规模工业增加值增速位居全省前列，旅游经济逆势上扬，远远领先全国平均水平，社会消费品零售总额增幅创15年以来新高，这是我市工业经济发展最强劲的一年，是我市旅游经济效益最突出的一年!
    第三，坚持统筹发展，扎实开展新农村建设，城乡面貌进一步提升。我们始终以新农村建设为目标，千方百计解决“三农”问题，实现农业总产值58亿元，增长6.2%。投入6亿元建设资金，继续推进“抓百村促千村”工程，新增新农村建设“十有”标准达标村46个。新增省级农业产业化龙头企业6家、农民专业合作组织66个；规模以上农产品加工企业实现营销收入34.59亿元，茶业综合产值突破30亿元，黄山区、徽州区被授予中国名茶之乡。大力改善农村生产生活条件，9万农村人口解决了饮水安全问题，行政村通公路率、通水泥（油）路率分别达到99.3%和89.3%，综合治理水土流失面积105平方公里，新建沼气池4000口，祁门、休宁、黟县被评为国家级生态示范区。全面加快区县城和村镇建设，完成投资88.4亿元，全市城镇化率达到39.8%。把壮大县域经济作为统筹城乡发展的主体工程，出台促进县域经济又好又快发展实施意见，县域经济和县域规模以上工业增加值增速分别高于全市1.1和3.5个百分点。进一步推进市中心城区建设，完成投资42.1亿元，安徽中国徽州文化博物馆、城市展示馆落成开馆，市城市总体规划修编和南部城镇群规划全面启动。过去的一年，我市农民人均纯收入连续第3年超过全国、全省平均水平，全国环境优美乡镇发展到7个，我市还荣获了省卫生城市、省人居环境奖城市和国家节水型城市称号，通过了创建国家环保模范城市省级预验收，这是新农村建设成果最丰硕的一年，是中国人居环境奖城市创建步伐最坚实的一年！
      第四，坚持改革开放，加快融入长三角，发展活力进一步增强。我们始终以开放提升影响力、以改革提高竞争力，积极参与泛长三角区域分工合作，在上海、杭州、厦门举办和参加了茶叶及名优农产品推介会、文化旅游产业对接会和投资贸易洽谈会，与上海光明集团、闽浙赣皖九方经济区分别就共建国家级东黄山旅游度假区、构筑区域大交通签署协议，组团赴港台、韩日等地开展招商、营销活动，全市实际利用外资1.3亿美元、到位内资161.1亿元，增长26.1%和37%；外贸进出口总额1.79亿美元，增长19.3%。着力办好重大节庆活动，吸引了来自23个国家的35个境外媒体和2800多名中外嘉宾。第四届中国国际徽商大会成功承办，第十二届黄山国际旅游节暨徽文化节放大了黄山对外效应，奥运火炬传递活动获得了“安徽第一、全国最好的城市之一”的评价。争取黄山口岸扩大开放获得突破性进展，恢复开通黄山至厦门、首尔等境内外航线。我市与瑞士因特拉肯市结为友好城市；黄山风景区与台湾阿里山签署了旅游发展合作协议、与希腊莱斯沃斯石化森林地质公园缔结为友好公园，旅游管理与可持续发展经验被联合国教科文组织和世界旅游组织誉为示范。我们以深化改革的实际成效纪念改革开放30周年。农村综合改革不断深入，集体林权制度主体改革基本完成。积极引入中国进出口银行开展业务并与国家开发银行签订了开发性金融合作备忘录，黟县、休宁农村合作银行挂牌成立，市国有资产运营公司正式组建。全市国有企业改制面达94%。非公有制经济增加值占生产总值的比重提高到59%，22家企业入选全省民营企业百强。过去的一年，我市跻身全省非公有制经济发展先进市行列，荣获2008浙商最佳投资城市特别奖和最佳国际休闲城市称号，成为全省唯一入选并蝉联世界特色魅力200强的城市，特别是成功举办了“三大活动”，这是我市会展节庆活动最出彩的一年，是我市深入推进改革开放最见成效的一年!
    第五，坚持以人为本，深入实施民生工程，人民生活进一步改善。我们始终把民计民生作为政府工作的根本任务来抓，认真组织实施20项民生工程，全市共投入资金7.2亿元，受益的城乡居民达118万人。加大就业再就业政策落实和工作力度，新增城镇就业1.5万人，帮助1万名城镇失业人员和困难群体实现再就业，小额贷款发放新增1200万元。加强社会保障体系建设，全市社会保障投入达15.1亿元，增长38.5%。全市城镇基本养老、医疗、失业、工伤和生育保险参保人数分别达10.24万、31.77万、10.6万、7.08万和3.89万人，企业退休人员养老金按时足额发放。全面完成农村低保提标扩面任务，城市低保对象实现应保尽保，城乡低保水平提高30%以上，农村“五保”对象集中供养率提高10个百分点，6497户“双困”家庭解决了廉租住房或租金补贴。城乡困难群众医疗救助实现全覆盖，101.9万农民参加新型农村合作医疗。“一卡通”发放财政各项补贴农民资金4亿元，受益农户达37.8万户。深入实施扶贫开发整村推进工程，全市投入扶贫资金6048万元，实施扶贫项目322个，8000人实现脱贫。投入资金3000万元，3181户雪灾和洪灾倒塌民房全部恢复重建。积极筹措1.01亿元资金，重点治理了65处省级地质灾害危险点。全市上下还捐赠款物逾4000万元，全力支援四川地震灾区抗震救灾。过去的一年，我市始终以高标准、严要求推进民生工程、开展“七保”工作，城镇登记失业控制率和农村低保覆盖面位居全省先进行列，特别是占全省一半以上的省级地质灾害危险点治理基本实现了“两个确保”的目标，这是我市民生工程投入最大的一年，是人民群众受益得实惠最多的一年!
    第六，坚持全面协调，不断加大公共投入，社会事业进一步发展。我们始终把社会建设与经济建设放在同等重要的地位，全市科教文卫体经费支出10.3亿元，增长37.5%。科技创新不断加强，全国科普示范县（区）创建活动扎实推进。大力实施知识产权战略，专利申请量达185件，9个产品被评为安徽名牌产品；新增省级企业技术中心2家，完成科技部数字景区服务示范工程，加快推进省制造业信息化工程重点市建设，被评为数字安徽五年建设先进单位。教育事业加快发展，义务教育经费保障机制进一步巩固完善，农村中小学D级危房改造任务全面完成，1.7万名中职学校家庭经济困难学生得到资助，普职招生比连续八年达到1∶1以上，位居全省前列。文化事业和产业发展进一步加快。徽文化艺术长廊、文化创意产业园等文化项目正式启动，屯溪老街被评为国家级文化产业示范基地；完成非物质文化遗产普查工作，新增9个国家级非物质文化遗产项目，又有4个村镇进入中国历史文化名镇名村，全市首批8家博物馆免费对公众开放。广播电视村村通工程年度任务全面完成。大力开展全民健身运动，国际山地车节、国际登山大会成功举办，在省第二届体育大会和特奥会上取得好成绩。公共卫生体系建设加快推进，手足口病防控和“问题奶粉”患儿诊治反应迅速、处置得当，市人民医院晋升为三级甲等医院。计划生育优质服务创建工作得到加强，人口自然增长率控制在3.03‰以内。认真开展城市标准化社区和示范社区创建活动，农村社区建设试点步伐加快，第七届村委会换届选举圆满完成。扎实推进平安黄山建设。“五五”普法深入实施。强化安全生产监管。突发公共事件应急管理体系进一步健全。扎实开展大接访活动，积极化解和妥善处理人民内部矛盾。严厉打击各类违法犯罪活动，社会治安防控不断加强，公安“三基”建设成效显著，奥运安保工作圆满完成，荣获2004～2007年度全省社会治安综合治理先进市称号。增进军政军民团结，国防动员、人民防空和后备力量建设进一步加强。妇女、未成年人、老年人、残疾人权益得到较好保障。统计、档案、地方志、气象、地震和民族、宗教、对台等工作取得新成绩。过去的一年，我市再次跻身全国双拥模范城行列，被评为安徽省首届文明城市，歙县获得全国文明县城称号，徽州文化生态保护实验区正式挂牌，社会治安综合治理工作在全省考评中位居第一，这是社会事业进步最显著的一年，是百姓安居乐业最祥和的一年!
    过去的2008年，也是政府自身建设进一步加强、施政能力进一步增强的一年。一年来，我们提升精气神、强化执行力，扎实开展政治生态环境建设年、反腐倡廉制度建设推进年活动,着力抓好以作风、行风为重点的“四大整治”，并取得阶段性成效。深化行政审批制度改革，修改、废止规范性文件17件和28件，取消、降低市管涉企经营性收费项目13项，市直90%的行政审批事项进入行政服务中心办理，现场办结率提高到80%。坚持依法行政，强化执法监督，及时修订市政府工作规则，规范行政处罚自由裁量权。积极执行政府信息公开条例，深入推进政务公开、村务公开和电子政务，“市民连线”公共服务平台办理群众来信来电1万余件。自觉接受人大及其常委会的法律监督、工作监督和政协的民主监督，交由政府系统办理的493件人大代表建议和政协提案全部办结。认真落实党风廉政建设责任制，强化审计监督和行政监察，严肃查处了一批违法违纪案件。
回顾过去的一年，我们也清醒地看到在发展与工作上存在的突出矛盾和问题。在经济建设上，主要是投资、产业、人才、城乡结构还不优、旅游业态创新还不够、工业园区特色化还不明显、农业产业化水平还不高，中心城市带动力和县域经济竞争力还不强，生态环保的工作压力还很大，经济发展方式也尚未得到根本转变。在社会建设上，城乡居民增收的难度加大，增加就业岗位、改善就医条件、提高就学质量以及困难群体住房保障、城市建设和社区管理等方面的问题较多，防灾抗灾、安全生产、市场监管等工作仍存在一些不足。在自身建设上，少数干部思想解放不够、发展意识不强，能力水平不高、服务观念不浓，研究谋划不深、工作作风不实，难以适应新形势新任务的要求。对此，我们一定高度重视，在更加扎实的工作中认真整改，努力解决。
    各位代表，我们能够在不平常的年份取得不平常的成绩，这是省委、省政府和市委坚强领导的结果，是市人大及其常委会和市政协监督支持的结果，是全市人民凝心聚力、真抓实干，抢抓大机遇、推进大开放、谋求大发展的结果。在此，我代表市人民政府，向全市广大工人、农民、知识分子、干部，向驻黄解放军指战员、武警官兵和政法干警，向各民主党派、各人民团体和各界人士，致以崇高的敬意！向关心、支持黄山现代化建设的中央、省驻黄单位和海内外友好人士，向在我市创业的投资者、建设者，表示衷心的感谢！

    二、2009年工作任务
    2009年，必将是一个非同凡响的年份：共和国诞辰60周年，邓小平同志视察黄山30周年，这是非常喜庆之年；国际金融危机的影响还在蔓延，困难和挑战还在加剧，这是非常艰难之年。对于我们这种综合实力不强、产业层次不高的地方来说，金融危机的滞后性影响会更大、持续时间会更长、不确定不稳定因素会更多，保持平稳较快发展的压力会更大。但我们必须看到，我国经济发展的基本面、所处的重要战略机遇期并没有改变，全省厚积薄发、加速崛起的阶段性特征也没有改变，我们已经构建了现代国际旅游城市建设与发展的良好格局，已经打下了实现平稳较快发展的良好基础，已经形成了心齐气顺、奋发有为的良好氛围。只要我们切实增强危机意识、发展意识、责任意识，坚决把握好调整变化中的一切积极因素，坚决利用好危机应对中的一切重大机遇，坚决发挥好日益凸显中的一切比较优势，就一定能争取黄山在新的历史起点上，开创新局面，再上新台阶!
今年政府工作的总体要求是：以邓小平理论和“三个代表”重要思想为指导，以科学发展观为统揽，全面贯彻党的十七大、十七届三中全会和胡锦涛总书记视察安徽重要讲话精神，认真落实中央、省经济工作会议和市委四届八次全会的决策部署，坚持以建设现代国际旅游城市为目标，以推进“十大工程”和“四区建设”为抓手，进一步强化大开放意识，增强大跨越信心，谋划大规划，推动大投资，建设大交通，发展大旅游，全力推进改革开放，全力优化经济结构，全力改善民计民生，全力维护社会稳定，加快“四大体系”建设，加快旅游国际化、新型工业化、农业产业化、城乡一体化进程，确保经济社会平稳较快发展。
今年经济社会发展的主要预期目标是：地区生产总值增长10.5%，财政收入增长13%；城镇居民人均可支配收入增长8%，农民人均纯收入增长7%；居民消费价格涨幅控制在4%以内；城镇登记失业率控制在4.5%以内；人口自然增长率控制在4.5‰以内；万元生产总值能耗下降4%，主要污染物排放总量控制在省政府下达目标以内。在执行中，必须坚持稳中求快、实中求好，做到能快则快、越快越好。
重点抓好以下五方面工作：

  （一）全力以赴确保增长、加快发展
实现平稳较快发展，必须紧紧抓住中央扩内需保增长政策的机遇，全面激发投资拉动力，强化工业支撑力，激活消费原动力。
第一，切实扩大有效投入。强力推进“十大工程”建设，扎实开展“重大项目建设年”活动，实现全社会固定资产投资334亿元，增长25%以上。
着力抓好大项目。今年重点要抓住548个投资额在1000万元以上项目建设。加快建设综合交通提速升级工程，掀起黄山交通建设新高潮。强力推进黄杭高铁、京福高铁、皖赣铁路扩能改造工程开工建设，同步做好城乡规划、设施体系、产业布局的调整提升工作。加速黄山机场改造和黄祁高速、宣黄高速公路建设，加快205国道以及322、218省道改造。加紧开工500千伏永丰输变电工程，加大推进水利、通信等基础设施建设力度。围绕国家出台的“双十”规划，着力抓好24个、总投资达295亿元的重大项目对上争取，扎实推进黄千、巢黄、砀祁高速公路、环黄山公路等重大项目前期工作。启动编制“十二五”规划，谋划一批现代基础设施、公共服务设施项目，以及旅游、文化、生态和矿产、水能、林木资源开发等优势产业项目，力争新增项目储备超2500亿元。
    全力抓好大建设。围绕构建新型城镇规划建设体系，扎实推进城乡建设管理提升工程，完成城市总体规划修编和市域南部城镇群规划编制，全市城镇和市中心城区投资达140亿元、50亿元，城镇化率提高1.6个百分点以上，在争创中国人居环境奖城市上取得新进展。加快市中心城区建设，按照“完善老城区、调整工业区、规划新城区”的总体思路，优化区域功能布局，全力构筑皖南中心城市框架。不断完善城市内部交通体系，抓好路网、停车场、公交首末站改扩建。加大保徽、改徽、建徽力度，抓好城市风貌整治，提升亮化、绿化、美化水平。加快市域南部城镇群污水处理、源水等重大公共基础设施共建共用共享，高标准规划新黄山铁路客运站，打造铁路、公路“零换乘”集散枢纽。加快区县城建设，大力培育旅游型、工业型、商贸型、城郊型、综合型小城镇，重点抓好20个特色城镇建设和发展。进一步推进市政公用事业市场化，建立数字化城管网络，提高城市管理整体水平。
    第二，切实强化工业支撑。坚持以工业经济为支撑、以工业园区为重点、以科技创新为动力不动摇，强力推进园区经济提速增效工程，促进“工业企业向园区集中、生产要素向重点产业集中、同类产业和相关产业向工业园区内的功能专业区集中”，力争新增规模以上企业60户，规模以上工业增加值增长25%。
加大工业扶持力度。全面落实支持工业企业抗风险保增长的政策措施。强化工业经济运行的监测、指导、服务、帮扶，组建全市中小企业服务机构联合体，多渠道帮助企业解决用工问题，市工业发展专项资金规模扩充到1000万元。鼓励企业实施知识产权战略，争创著名商标和名牌产品。着力抓好规模以上企业发展，重点支持永佳集团等企业做大做强。指导企业用足用好进出口促进政策，扩大先进技术装备和资源性原材料进口，增强机械电子、纺织服装、农特产品、精细化工四大板块的国际市场竞争力，实现外贸进出口总额2亿美元，增长12%。
加快园区发展步伐。编制实施全市园区产业发展总体规划，制定出台特色园区发展政策，引导各园区明确产业定位及发展取向，坚持“三个围绕、一个不上”，推进“异地办厂、原地纳税”，集约发展比较优势产业，在打造徽州区精细化工园、市文化创意产业园上率先突破。着力培育文化产品、农特产品、旅游商品等特色产业，力争增加值分别增长22%、26%、30%。落实建设用地投资强度和容积率“双控”标准，提高土地利用综合效益。加强基础设施建设，大力发展生产性服务业。力争全市园区完成投资53亿元，实现规模以上工业增加值27亿元，税收收入3.7亿元，其中市经济开发区分别达到12.5亿元、3亿元、0.5亿元。
    第三，切实激活城乡消费。加大争取国家服务业综合改革试点城市工作力度，认真编制实施服务业发展规划及配套政策措施，确保全年社会消费品零售总额达到104亿元，增长15%。合理布局、丰富发展徽菜美食、茶楼酒吧、娱乐购物等服务业态，结合黎阳老街改造、商业步行街建设，培育一批有特色、上规模、高品位的商业街区。依托综合交通运输体系，规划建设区域性货运站场、物流配送中心和大型批发市场，发展电子商务。鼓励和规范商贸企业促销活动，积极引进知名零售集团。推进城市社区“双进”工程，抓好屯溪区阜新社区国家级商业示范社区建设，全面开展屯溪区省级城市商贸服务示范区试点。深入推进万村千乡市场工程，加快农村流通网点建设，发展标准化乡村连锁农家店，组织好家电下乡、农机下乡，拉动农村消费。全面落实促进住房消费的政策措施，促进房地产市场持续健康发展。

    （二）矢志不移坚持特色、强化优势
坚持走旅游发展、环境保护、文化传承“三位一体”的特色优势发展道路，坚决贯彻省委、省政府“提升以黄山为中心的皖南地区开放开发水平，着力打造皖南旅游文化示范区，加快建设吸引力更强的世界级旅游目的地”的战略方针，按照带动辐射、互补多赢、整体提升的大旅游发展思路，积极联动周边，制定实施方案，争取政策支持，加快启动和建设皖南旅游文化示范区项目。
    第一，提升旅游产业上水平。大力实施旅游综合开发提升工程，全年接待游客2070万人次，旅游总收入162亿元，各增长15%。
以开发建设新业态为增长点。巩固提升一批传统观光旅游品牌，全力支持、深入推进黄山风景区综合开发，精心打造国际精品旅游景区。大力发展休闲度假、文化体验、商务会展、康体健身等新兴业态，加快实施新安江、太平湖综合开发以及雨润、东黄山、奇墅湖度假区(村)等重点项目建设，争取引进1～2个国际知名酒店品牌，确保完成投资48.6亿元。继续推进中法乡村旅游合作等项目，加快11个片区、36个主题村落的差异性开发，打造一批星级农家乐、旅游示范村、特色乡村酒店，力争西递、宏村进入5A级景区行列。以中国生态旅游年为主题，组合推出高尔夫、温泉、户外运动等专项旅游产品。组织开展首届徽州工艺大赛、展销及“十大徽匠”评比，着力培育一批旅游商品生产企业、拳头产品和专业市场。
    以整体谋划新营销为突破点。坚持“政府引导、企业主体、上下联动、市场运作”，推动城市形象和旅游产品的整体营销。全面开拓国内旅游市场，积极培育冬季、非周末和散客市场。加强徽文化与佛文化的联动、山水旅游与红色旅游的联动，热推“大黄山”、“古徽州”精品线路和“名城-名湖-名山”世界级黄金旅游线。加大台湾市场营销力度，巩固韩日和港澳等传统市场，开拓欧洲及俄罗斯等新兴市场。加大创意力度，进一步打响黄山国际旅游节暨徽文化节品牌，进一步办好山地车节、健走节、摄影大赛、登山大会和“黄山论剑”等活动，吸引全球目光，扩大国际影响。
以配套完善新环境为着力点。制定实施支持旅游企业度难关、抢市场的政策措施。构建集营销、服务、管理于一体的旅游信息化网络。抓好旅游服务标准化建设，加强旅游联合执法，健全长效监管机制。加快主要景区（点）循环路网建设，建成市旅游集散中心二期工程，扩大旅游公交网络；建设自驾游配套服务设施，完善停车、标识、资讯、应急、救援系统。发挥旅游行业协会自律作用，加强从业人员队伍建设，提高市民文明素养，抓好以屯溪老街“徽商诚信街”为龙头的诚信建设活动，打响“诚信经营”、“诚心服务”的新徽商品牌。
    第二，加强生态环保上水平。大力推进生态环境保护建设工程，力争进入国家环保模范城市行列。
    扎实抓好节能减排。严格落实节能减排工作责任制。积极推广新技术、新装备，抓好10户重点企业和化工等重点行业的节能降耗。建成覆盖全市区县城的生活污水处理厂和垃圾集中处理场，推进重点乡镇环境基础设施建设，确保城市污水处理率、垃圾无害化处理率分别达85%、95%以上。加快城市和旅游景区照明节能改造，严格执行建筑节能强制标准，确保符合节能设计标准的建筑面积达到30%以上。深入实施生态家园富民工程，强化畜禽养殖污染综合整治，新建沼气池4000口以上。编制实施循环经济重点专项规划，确保1～2个区县、力争1～2个园区进入全省试点；完善废旧物资回收、加工、利用体系，发展再生资源产业。落实最严格的耕地保护制度和节约用地制度，力争完成全市土地利用总体规划修编，用好土地置换政策，加强用地批后监管，建立健全闲置土地处理长效机制。
    继续抓好生态建设。加强风景名胜区、自然保护区和森林公园、地质公园、湿地公园的保护与管理，推进齐云山申报世界自然遗产工作。实施松材线虫病三道防线建设，抓好林政管理、森林防火和野生动植物保护工作，巩固荒山造林、退耕还林、封山育林和生态公益林建设成果。加快推进新安江流域生态补偿机制试点，继续抓好月潭水利枢纽工程建设前期工作。落实建设项目生态恢复措施，坚持不懈地做好地质灾害防治工作，综合治理水土流失面积90平方公里。大力加强饮用水源地保护和河道采砂管理，解决8万农村人口饮水安全问题。切实抓好城市环境的综合整治。
第三，壮大文化实力上水平。大力弘扬徽文化特色优势，加快推进文化产业精品打造工程和城市文明提升工程。
    着力发展文化产业。促进文化与旅游更紧密融合，深化文化体制改革，出台文化产业发展政策，组建文化投资公司，引导和鼓励社会资本发展文化产业。推动市级文化产业示范基地建设，着力抓好25个重点文化产业项目，力争完成投资15亿元。加快建设徽文化艺术长廊，规划发展特色博物馆群、文化艺术创作基地、“徽州四雕”生产展销基地和文化娱乐产业基地。以市场化方式推出地方特色的旅游定点演出。深入开发屯溪老街等区域文化资源，拓展文娱休闲功能；推进徽州府衙恢复工程建设，打造徽州古城品牌。抓好文化创意产业园项目。力争全市文化产业增加值占生产总值比重达到7.5%。
繁荣发展文化事业。编制实施徽州文化生态保护实验区总体规划，严格落实黄山市文物保护管理办法，加大文物、物质和非物质文化遗产保护力度，加快推进“双千”、百名艺人传承、徽州文化记忆工程，积极做好徽墨制作技艺申报世界非物质文化遗产工作。深入挖掘徽州文化，丰富、活跃徽学研究。出版发行《黄山市志》。继续做好徽州历史档案抢救保护工作，开工建设市档案馆。举办全国新安画派精品展，开展“百名画家画黄山”活动。建成市美术馆、徽剧馆、歌舞剧团工程。不断完善城乡公共文化服务体系，积极开展送戏下乡进社区和文化广场活动，抓好网吧、歌舞厅等娱乐场所文化市场管理。积极发展新闻出版、文学艺术和哲学社会科学事业。广泛开展全民健身运动，举办市第五届运动会，备战省第十二届运动会。深入开展公民道德践行活动，深化文明创建城乡结对共建工作，切实解决创建工作薄弱环节，切实加强乡风文明建设，切实提升文明城市创建水平。

    （三）千方百计稳固基础、促进增收
坚持把解决“三农”问题作为政府全部工作的重中之重来抓，按照新农村建设“八个一批”的要求，深入实施新农村建设巩固拓展工程，在农业增效、农民增收、农村发展上实现新突破。
第一，以实施“1122”计划为重点，大力发展农业产业化。完善全市农业产业化规划纲要和相关产业集群规划，设立农业产业化发展专项资金，培植十大产业基地，培育十大龙头企业，争创20个知名品牌，争上20个重大项目。围绕发展壮大主导产业，切实抓好基地建设和规模化生产，加快实施生态茶园“双十”工程、养殖业“451”工程和林业产业提升工程，建成高效生态茶园5万亩和毛竹、干鲜果、蚕桑、中药材、商品蔬菜、花卉苗木3万余亩，大力发展油茶等木本油料产业。推进农业产业化“125”提升行动，力争超亿元、超5000万元的龙头企业分别达到6家、17家，农民专业合作组织发展到400个以上。推进标准化生产，抓好动物疫病防控和农产品质量安全全程监控，尽快建成国家级茶叶检测中心，新增省级农业标准化示范基地3～5个、“三品”认证面积4.5万亩以上。加大品牌建设力度，积极申报祁门红茶、顶谷大方、五城茶干地理标志产品，争创省著名商标、名牌农产品5～10个。办好黄山茶叶及名优农产品交易会等促销活动。加快农业技术服务体系建设，推进科技入户工程、科技特派员基层创业行动、科技专家大院活动，科技进步对农业增长的贡献率提高到50%以上。
第二，以完善农业发展机制为保障，全面加快农村改革进程。落实“三个大幅度”的强农惠农政策，力争全市财政对农业农村投入增长25%以上。稳定和完善农村基本经营制度，按照“三个不得”和依法自愿有偿原则，推进土地流转，力争2～3年内，全市规模经营比重提高10个百分点。深化农村综合改革，加强农村公共服务体系建设，建立健全县、乡、村三级为民服务全程代理网络。进一步完善集体林权制度改革，力争林权流转量达到全市集体山场的15%，促进林业结构调整和现代林业发展，发挥和利用好山区资源优势。开展新型农村金融试点改革，建立政策性农业保险制度，切实加大金融保险对农村改革发展的支持力度。
第三，以改善生产生活条件为基本，深入推进新农村建设。今年要抓实改善农村生产生活环境的基础性工作，把新农村建设与重大项目建设、村级规模调整与村庄规划提升有机结合起来，推进老村撤并整合、新村集中建设和移民统一安置，推进城乡结合部和景区周边农村率先一体化发展，加快建设一批示范村，新增“十有”标准达标村、基本达标村各50个。积极创建环境优美乡镇、生态文明村，全面开展“三清、三治”和“一池三改”行动，实施农村清洁工程，抓好村容整治和白色污染、河道垃圾清理。完善改造农村电网，硬化“村村通”公路480公里。推进小水电小水利建设，除险加固小型病险水库31座，提高防汛抗旱和气象防灾减灾能力。加快实施广播电视村村通等重点文化惠民工程和农民体育健身工程，新建乡镇综合文化站41个、农家书屋174个。建成标准化乡镇卫生院19所、村卫生室95所。继续改善农村中小学办学条件，力争全市每个乡镇都建有一所寄宿制学校。

   （四）坚持不懈增强动力、激发活力
以纪念邓小平同志视察黄山30周年为动力，推进黄山新一轮改革开放，为地方经济社会发展注入生机和活力。
    第一，全力扩大开放和招商引资。积极准备对接2010年上海世博会，提前谋划利用2011年黄杭高铁建成通车后的“同城”效应，在融入长三角城市群和杭州都市圈，参与泛长三角区域分工合作上迈出更大步伐。推进闽浙赣皖九方经济区的互动发展，深化与珠三角、环渤海等地区的交流合作。精心组团参加第四届中部博览会、第五届徽商大会。力保黄山口岸扩大开放，培育开拓境内外新航线。加强对外友好交往，密切友城友山关系，做好外事侨务和台湾事务工作。要把扩大开放的重点放在招商引资上，必须抓得紧而又紧、实而又实，确保全年引进内资193亿元，实际利用外资1.43亿美元，分别增长20%、10%。创新招商方式，完善招商机制，推动专业招商、突出以商招商、发展代理招商、鼓励各方招商，发挥开发区、企业和小分队主体作用。优化招商环境，加快完善产业配套能力，切实提升服务水平。夯实招商基础，做好项目规划编制工作，建立知名企业信息库，促进招商引资向招商选资转变，力争在世界500强企业、台湾地区和长三角地区招商上取得新突破。
    第二，强力推动改革与科技创新。继续推进行政审批制度改革，着力抓好行政服务中心建设，市直行政审批事项现场办结率提高到85%、平均对外承诺时限缩减为5个工作日以内。稳步推进事业单位分类改革。加大企业改革攻坚力度，妥善解决改制企业遗留问题。建立健全自主创新激励政策，完善科技投入稳步增长机制，积极参与对接合芜蚌自主创新综合配套改革。鼓励产学研合作，落实与浙大、合工大、中科大的合作协议，支持永佳集团建设科技企业孵化器，力争新增国家级、省级企业技术中心1家和2家，组织实施亿元以上重点技改项目7项，力争企业技改投入突破35亿元。完善信息基础设施建设，构建数字黄山的基本框架。全面启动人才强市战略推进工程，抓好实用急需人才、高层次高技能人才和特色人才队伍建设。完善人才资源信息库和人力资源市场体系，鼓励引资与引智、引进与引用相结合，发挥黄山籍旅外人士的积极作用，扎实办好博士后科研工作站，构建皖南人才高地。
    第三，着力强化财政与金融保障。抓住当前积极的财政政策和适度宽松的货币政策机遇，全力争取民生工程、基础设施、生态环境、主体功能区建设等方面政策资金支持，引导金融机构增加有效信贷投放，确保新增贷款增幅超过全省平均水平。切实抓好增收节支，提升财政运行质量。推进增值税转型改革，深化部门预算、国库集中支付、政府采购、非税收支改革。依法加强税收征管，落实结构性减税政策，着力培植新的增长点和骨干财源。调整优化支出结构，总体严控行政性支出，坚决压缩一般性开支，严格接待登记、回访督查制度，抓好公车定编、公务卡试点，集中资金保障重点投入。加大金融支持力度，加强政银企对接，提高金融服务实体经济水平。大力引进金融机构，优化银行县域网点布局，新组建农村合作银行、小额贷款公司2家和7家以上，积极争取村镇银行试点。抓好市国有资产运营公司市场化、规范化运作。落实与国家开发银行的新一轮合作，进一步发挥城市建设融投资的功能作用。加强担保机构建设，鼓励加入省担保集团再担保体系，全市担保机构发展到20家，市丰元担保公司年累计担保责任总额超2亿元。鼓励上市公司再融资，加快泰达、好视达公司上市步伐。
第四，大力发展县域和民营经济。进一步突出县域经济在全市经济发展中的地位，按照省、市先后出台的促进县域经济发展的政策措施，结合实际，创新方法，抓好落实。强化市直部门服务县域经济发展的职责作用，促进省扩权试点县用足用活有关政策，其他区县比照试点县享受市级扩权等政策，确保县域经济增幅高于全市平均增幅。以省试行分类考核为契机，发挥全市县域、镇（乡）域经济考核体系的导向作用，加大奖惩力度，促进县域发展在全省争先进位。坚持把民营经济作为县域经济的主体来抓，出台实施非公有制经济发展政策措施，完善政府与民营企业家联系制度，表彰奖励一批发展民营经济的先进典型，大力营造亲商、安商、富商的良好氛围。加强对已取消行政审批和收费项目的后续监管，落实涉企处罚报知制度，切实减轻企业负担。加快建立社会化服务体系，培育和发展各类行会、商会，引导和支持民营企业加强制度和科技创新，培养和造就一批新徽商群体、企业家团队，力争全市非公有制经济增加值占生产总值的比重提高1.5个百分点以上。

  （五）扎实有效改善民生、共建和谐
    坚持以人为本、以民生为重，发展社会事业，强化就业保障，完善社会管理，在努力解决好维护好民计民生的基础上，建设和谐美好黄山。
    第一，实施民生改善工程。为多办民生实事，多解决群众困难，多让老百姓得实惠，市里今年在省确定的28项重点民生工程建设任务的基础上，再增加菜篮子工程和精神病患者防治2项民生工程；在全市投入7.4亿元用于省市民生工程的基础上，再增加1370多万元，实施“春风行动”，重点解决困难群体“就业就医就学”突出问题，尽最大努力让特困家庭人员能就业、能看病、能上学！
第二，发展教育卫生事业。坚持教育优先发展，完善义务教育免费政策和经费保障机制，全面建立各个教育阶段学生资助体系。依法逐步均衡配置公共义务教育资源，加快义务教育阶段标准化学校建设，提升现代远程教育质量，认真实施课程改革，推进素质教育发展。做好农村留守未成年人教育工作。加快高中教育普及进程。整合资源、多方发展职业教育，加大争创国家级重点职校和区县职教中心建设力度，积极筹建职业技术学院。支持黄山学院扩建升级。完善公共卫生体系，继续推进城乡卫生服务网络规范化建设。传承新安医学，促进中医事业发展。加强医疗卫生人员医德医风建设，提高医护服务质量和水平。强化食品药品安全监管。大力开展爱国卫生运动，争创国家卫生城市。推进计划生育技术服务机构标准化建设，完善农村部分计划生育家庭奖励扶助制度，抓好关爱女孩、出生人口性别比综合治理以及城市和流动人口计生管理服务工作，提高妇女儿童健康水平。
                  
    第三，完善就业和社会保障体系。认真落实省“促进就业23条”，实施更加积极的就业政策，深入开展就业援助和就业服务，统筹做好返乡农民工、高校毕业生、下岗失业人员就业和退役军人安置工作，确保新增城镇就业1.5万人、就业困难人员再就业1900人。千方百计增加就业岗位、创造就业机会，鼓励发展劳动密集型产业，继续抓好创业园、创业街建设，支持自主创业和自谋职业。广泛开展新型农民培训和农民工技能培训，抓好用工对接和信息服务，促进返乡农民工就近就业、就地创业。努力增加劳动者收入，落实义务教育学校实施绩效工资意见，提高企业退休人员基本养老金，健全劳动合同和劳动用工登记制度，建立企业职工工资支付保障机制，确保农民工工资发放。加大社会保险扩面征缴工作力度，城镇基本养老、医疗、失业、工伤、生育保险参保人数分别达到10.5万、28.5万、10万、7.2万和4万人，基金征缴率达95%以上。全面落实被征地农民养老保险政策。加强农村“五保”供养服务机构建设，提高城乡低保规范化水平。建立城乡一体化的医疗救助绿色通道，新型农村合作医疗参合率提高到93%。加大廉租房等保障性住房建设力度。深入开展“七保”工作。抓好边远山区库区老区扶贫开发，加快整村推进工程，落实新的扶贫标准，确保6000人稳定脱贫。发展残疾人事业，做好重度残疾人生活救助等工作。整合利用现有资源，加强老年人和青少年活动设施建设。强化生活必需品市场监管，确保正常供应和价格基本稳定。
    第四，加强社会管理工作。深化社区管理体制改革，扎实开展城市社区居民自治提升年和农村社区示范点建设。支持工会、共青团、妇联等人民团体及志愿者队伍广泛参与社会管理及公共服务。扎实做好新形势下的民族宗教工作。巩固发展军政军民团结，落实优抚政策，加强国防教育、国防动员和民兵预备役及人防建设，夯实全国双拥模范城“三连冠”工作基础。深入开展“安全生产年”活动，全面落实安全生产责任制，加大重点行业及领域的隐患排查治理和重大危险源防控力度，严防重特大事故发生。深入开展“五五”普法，拓展法律援助和法律服务。进一步增强信访工作的预见性、主动性，畅通群众诉求渠道，提高矛盾纠纷发现率和调解成功率，积极预防群体性事件。加强市和区县应急指挥体系建设，提高应对处置突发公共事件的能力。强化社会治安综合治理，加强公安基层基础工作，力争所有乡镇（街道）建立综治工作中心，所有社区和重点村建立警务室。防范和打击各类违法犯罪活动，做好刑释解教人员帮教安置和社区矫正工作。确保1～2个区县进入“平安县”行列，确保群众安全感继续保持全省领先。三、切实加强政府自身建设做好今年各项工作，确保经济社会平稳较快发展，任务非常艰巨、政府责任重大。我们必须深入学习实践科学发展观，切实提高领导科学发展和驾驭复杂局面的能力，建设一个无愧于使命、让人民满意的政府。
    第一，真抓实干促发展。千事万事抓科学发展是大事。抓发展是政府工作的第一要务，必须不动摇、不懈怠、不折腾，必须求真务实、真抓实干。真抓实干，就是要解放思想抓发展。思想解放的深度，决定改革开放的程度，决定科学发展的速度。面对新机遇、新挑战、新任务，必须要有新思路、新举措、新办法，一定要善于学习、善于研究、善于谋划。解放思想，最重要的是要有尽职尽责的态度、创新创业的精神、开明开放的胸怀和敢闯敢试的胆识，一心想着发展，一切为了发展，勇于超越做改革的促进派，勇于突破做创新的实践者，勇于争先做发展的先行官，不讲不能做、不好做，只讲怎么做、怎么做得更好，让整个政府的工作更有生气、更具锐气、更为大气。真抓实干，就是要脚踏实地抓发展。凡是属于政府该办的事、该政府办好的事，必须雷厉风行，马上做、认真做；凡是那些长期积累形成而又需要一定过程去解决的事情，必须锲而不舍，一步一步攻坚，一点一滴化解；凡是那些带有全局性质而非局部能够解决的事情，必须大胆探索，积极地尝试，稳妥地推进。坚决避免有想法没办法、有上文没下文的现象；坚决克服讲得到做不到、说得好干不好的问题。在今年困难多、任务重的情况下，政府各个部门和工作人员一定要盯紧目标抓发展、围绕大局抓发展、突出实效抓发展，真正做到以实绩论奖惩，以发展论成败。
    第二，倾心尽力为人民。千要万要为人民谋利益最重要。为人民是政府行政的最高宗旨，必须办实事、做好事、解难事，必须真情实意、倾心尽力。倾心尽力，就是要把勤政为民的理念融注入思想情感里。我们一定要在思想上尊重群众，在感情上贴近群众，始终把群众当作自己的亲人，时刻把百姓的冷暖挂在心头，群众的必需就是政府要抓住的大事，群众的疾苦就是政府要化解的难题，群众的关注就是政府要努力的方向。要想民所想、忧民所忧、急民所急，从群众的角度出发、为群众的利益考虑，真正做到权为民所用、情为民所系、利为民所谋。倾心尽力，就是要把勤政为民的情感体现到政策措施上。必须完善科学民主决策机制，问政于民、问需于民、问计于民，深入基层体察民情、深入一线了解民意，切实提高政策措施的针对性、具体性和实效性，避免因决策失误和工作不当损害群众的利益、伤害群众的感情。政府任何一项政策、一项措施，只要出发点、落脚点是为了黄山的发展，为了人民的利益，即使有再大的困难、再大的风险、再大的责任，我们都有理由以巨大的勇气和魄力去实践、去担当。倾心尽力，就是要把勤政为民的措施落实在具体工作中。政府工作要在具体中深入，在深入中落实。群众利益无小事，再小也要用心办、细心办、耐心办；政府工作无戏言，凡是对人民的重托、对基层的表态、对投资者的承诺，一定要掷地有声、言而有信、坚决兑现。我们必须继续扎实推进“四大整治”活动，必须完善工作机制和推进流程再造，必须加大督查督办和责任追究。我们的政府工作人员一定要以夜不能寐、食不甘味的劲头抓工作，政府各项工作一定要更有章法、更有节奏、更有成效，政府部门服务群众、服务基层、服务企业的效能和水平一定要有新的改进、新的提高。
    第三，风清气正树形象。千好万好要把政府的队伍建设好。树形象是政府建设的集中体现，必须讲正气、讲法纪、讲团结，必须率先垂范、风清气正。风清气正，就是要从严律己、拒腐防变。政府各部门及其工作人员，一言一行都体现着政府行为、一举一动都代表着政府形象，必须对自己高标准、严要求，决不因小事而不为、决不因事小而不严。市政府班子更要在政治生态建设上做好样子带好头，要求别人做的自己先做到，禁止别人做的自己坚决不做，自警自励、慎独慎微，真正成为干事创业的模范、清正廉洁的表率。同时，在推进惩治和预防腐败体系建设上毫不懈怠，在严肃查处违纪违法的人和事上毫不手软。风清气正，就是要依法行政、规范行为。我们必须恪守权由民授、权必制约的法治理念，自觉接受人大及其常委会的法律监督、工作监督和政协的民主监督，认真办理人大代表议案建议和政协提案，虚心听取民主党派、工商联、无党派人士和各人民团体的意见。必须充分保障人民群众的知情权、参与权、表达权、监督权，深入贯彻落实加强市县政府依法行政的决定和政府信息公开条例，办好电子政务和“市民连线”、“政风行风热线”，广开言路、广纳谏言，让权力始终在阳光下、在法制的轨道上运行。风清气正，就是要珍视团结、形成合力。政府要有所作为、大有作为，靠的是整体效应，靠的是团结奋斗。站得高、看得远才能谋团结，想干事、干成事才会求团结，对人宽、对己严才是讲团结。作为一个团队，我们必须倍加珍惜共事的缘分、干事的机会，工作中既要坚持民主，又要善于集中，既要本位思考，也要换位思考，既要摒弃私心，更要出于公心，真正做到相互宽容、相互理解，相互补台、相互支撑，坚决做到同事同心、干净干事，与广大人民群众共克时艰、共创辉煌。

    各位代表，古老的徽州曾经写下经济繁荣、名垂史册的华丽篇章，今天的黄山正在描绘大发展、大跨越的更新更美蓝图。困难激励斗志，挑战唤发激情。让我们在中共黄山市委的坚强领导下，把人民的期望化为前进的动力，把发展的使命变成积极的行动，为全面完成今年的各项目标任务，为推进“十大工程”和“四区建设”，为加快现代国际旅游城市建设而团结拼搏，以优异成绩向新中国成立60周年献礼！
